# Effect of waste cooking oil on the performance of EVA modified asphalt and its mechanism analysis

**DOI:** 10.1038/s41598-024-64816-9

**Published:** 2024-06-18

**Authors:** Renwei Zhang, Junfang Wang, Haixin Kang

**Affiliations:** 1 School of Architecture and Civil Engineering, Sanming University, Sanming, 365004 Fujian China; 2 Key Laboratory of Engineering Material & Structure Reinforement in Fujian Province Colleges (Sanming University), Sanming, 365004 China

**Keywords:** EVA modified asphalt, Waste biological oil, Performance evaluation, Mechanism analysis, Engineering, Materials science

## Abstract

The balance between the low and high temperature performance of asphalt materials is important to avoid either rutting deformation or low temperature cracking resistance of asphalt pavement. This is beneficial for improving the asphalt pavement comprehensive performance. Considering the excellent high temperature performance of Ethylene–vinyl acetate (EVA) modified asphalt, this study first modified it with Waste Biological Oil (WBO) to prepare WBO/EVA composite modified asphalt (WEMA) with different dosages. Then the samples were evaluated by the traditional physical properties, low and high temperature rheological properties. Finally, the micro mechanism of WBO on EVA modified asphalt were explored by gel permeation chromatography (GPC) test and atomic force microscope (AFM) experiments. The experimental results reveal that WBO has a softening effect on EVA modified asphalt, reducing its stiffness and improving its stretching performance and flowability. In addition, WBO can reduce the high-temperature deformation resistance of EMA modified asphalt, but it significantly enhances the low-temperature property of EVA modified asphalt. When the WBO content ranges from 1.5 to 2.5%, the high-temperature performance of WEMA is inferior to that of EVA-modified asphalt, however, its low-temperature performance is significantly better than that of EVA-modified asphalt. Importantly, within this WBO content range, the comprehensive performance of WEMA is superior to that of pure asphalt. Mechanism investigation showed that WBO reduces the content of macromolecular micelles and average molecular weight in EVA modified asphalt, and it also diluts the asphaltene components in the asphalt system, resulting in a slight weakening of the performance of WEMA at high temperatures and a significant performance enhancement at low temperatures. Ultimately, the utilization of WBO/EVA composite modified asphalt has a better comprehensive performance.

## Introduction

The rapid development of transportation infrastructure still maintains a high demand for asphalt materials. At the same time, with the development of the social economy, the significantly increased traffic volume and load have required better performance of asphalt^1–3^. Modifying asphalt is one of the most effective ways to improve its performance, and polymer modifiers reveal outstanding reinforcing extent on the asphalt property^4–6^. As a result, they are widely used in asphalt modification research. Common polymer modified materials namely styrene butadiene styrene (SBS), polypropylene (PP) and polyethylene (PE) have faced challenges in forming a stable and compatible system with the asphalt in the engineering practices^7,8^. When polymer modified asphalt is stored under high temperature conditions, the polymer may be separated from the pure asphalt due to insufficient compatibility between the polymer modifier and the pure asphalt. For example, although researchers have improved the compatibility between the most widely used polymer modifier and asphalt by adding different stabilizers and strictly controlling the preparation process, the insufficient compatibility between polymer modifier and the pure asphalt is still one of the objective factors limiting its production and construction application^9^.

Ethylene–vinyl acetate (EVA) is a thermoplastic polymer that can crosslink three dimensionally with the pure asphalt under appropriate conditions. The EVA can improve its compatibility with asphalt by inserting polar groups into polyolefins, which could help to generate a more stable polymer network. In addition, the ester groups in EVA can strengthen the polarity of molecular chains and decrease their crystallization ability, thereby improving the chemical suitablity between polymers and asphalt^10,11^. Champion et al. investigated the hardening mechanisms of asphalt modified with EVA and other types of polymers. The results showed that the cracks in EVA modified asphalt developed at the interface of the two phases, exhibiting brittle behavior. Bulatovic et al. studied the rheological properties of EVA-modified asphalt and found that EVA reduced the low-temperature crack resistance of pure asphalt. The drawback is that although EVA could obviously improve the pure asphalt at high temperatures, it has a certain negative effects on its performance at low temperature, which limits the prospects of promoting the use promotion of EVA modified asphalt in engineering practices.

In recent years, with the increasing attentions in the global environmental protection field, more and more researchers have focused on the utilization of renewable resources. Bio-oil is a renewable resource prepared from biomass, which includes crops, wood, and animal waste^12–14^. The popularization of environmentally friendly concepts and the advancement of industrial technology have promoted the adoption of bio oil in the asphalt field^15,16^. Fini et al. explored the impact of different bio-oils on asphalt property, and indoor tests showed that bio-oil modified asphalt has satisfactory low temperature crack resistance^17^. Han et al. considered the advantages of bio-ash modified asphalt and bio-oil modified asphalt, and used bio-ash and vegetable oil to prepare a biological asphalt with outstanding low and high temperature performance^18^. Zargar et al. investigated the potential of waste biological oil (WBO) as a rejuvenator for asphalt being aged and found that WBO can significantly reduce its softening point value. When the content of WBO is 3%, it can make the value of softening point of asphalt being aged to its original unaged status^19^. Gao et al. investigated the rheological properties and property sensitivity of asphalt modified by bio oil derived from wood, and the results showed that bio oil have a reducing effect on the deformation ability of asphalt^20^. With the increase of content of bio oil, the crack resistance slightly improves, softening point value and the rutting resistance of unaged samples decreases. Sun et al. conducted indoor experiments to study the influence of waste edible oil on the chemical characteristics and physical performance of asphalt. Bio oil reduces the viscosity, and improves the permeability and ductility of asphalt samples^21^.

Given the excellent high-temperature stability and poor low-temperature property of EVA modified asphalt, it is necessary to consider the combined use of WBO to obtain modified asphalt with both excellent high-temperature deformation resistance and low-temperature deformation performance. Therefore, on the basis of the above viewpoint, this article uses WBO to modify EVA modified asphalt (EMA) and prepares WBO/EVA composite modified asphalt (WEMA). Then, rheological properties of WEMA at low and high temperature and the influence of WBO on the comprehensive property of EVA modified asphalt are explored. Finally, gel permeation chromatography (GPC) test and atomic force microscope (AFM) test are used to deeply analyze the influence of WBO on the molecular weight and microscopic morphology of EVA modified asphalt, so as to reveal the important microscopic modification mechanism.

The objective of this study is to prepare modified asphalt with both excellent high-temperature and low-temperature performance by using EVA-modified asphalt with superior high-temperature performance and renewable WBO resources. Additionally, the study aims to investigate the important modification mechanisms. The research findings can provide asphalt materials with good comprehensive performance for asphalt pavements, aligning with the concept of sustainable development in road materials.

## Materials and methods

### Test raw materials

This article uses 70 # pure asphalt and EVA modifier to prepare EVA modified asphalt. The WBO used is peanut oil (dark yellow liquid) discarded after being used in the kitchen. Pure asphalt is bought from Jiangsu Tongsha Co., Ltd., and EVA powder is obtained from Huachuang Plasticization Co., Ltd. Table 1 shows the basic performance parameters of the test raw materials.

**Table 1:** Basic property parameters of original testing materials.

Material	Test parameter	UNIT	Value
Pure asphalt	Penetration at 25 °C	dmm	65.0
	Softening point	°C	47.5
	Viscosity at 135 °C	Pa s	0.41
	Ductility at 10 °C	mm	33
EVA	Density	g/cm^3^	0.948
	Mass flow rate of solution	g/10 min	4.0
	Tensile yield strength	Mpa	2.94
	Bending modulus	Mpa	6.86
	Elongation at break	%	700%
WBO	Relative density	–	0.9110–0.9180
	Freezing point	°C	0–3
	Fatty acid content	%	94.96

### Modified asphalt preparation

Firstly, EVA modified asphalt is prepared, and then WBO is mixed with EVA modified asphalt to prepare WBO/EVA composite modified asphalt. The specific preparation steps are divided into three steps. Step 1:5% (mass fraction) EVA modifier is added into the pure asphalt at 175 ± 5 °C, according to the previous studies, and then a high-speed shearing machine is used for shearing^22^. The shearing rate and time is set as 5000 r/min and 0.5 h, respectively. Step 2: WBO with different dosages (0.5%, 1%, 1.5%, 2%, and 2.5%) is added into the EVA modified asphalt. Then, the high-speed shearing machine is used for shearing for 0.5 h at 170 ± 5 °C, and the speed is maintained at 5000 r/min. Step 3: The modified asphalt is put into an oven at 165 °C for 4 h to swell, and it is stored for later use.

In order to facilitate the analysis and discussion of the results, the asphalt samples are named by different labels, and the labels are shown in Table 2.

**Table 2:** Design scheme of modified asphalts.

Asphalt label	Modified asphalt composition
BA	Pure asphalt
E5	5% EVA modified asphalt
EB05	5% EVA + 0.5% WBO modified asphalt
EB10	5% EVA + 1.0% WBO modified asphalt
EB15	5% EVA + 1.5% WBO modified asphalt
EB20	5% EVA + 2.0% WBO modified asphalt
EB25	5% EVA + 2.5% WBO modified asphalt

### Experimental procedures

**Figure 1:**
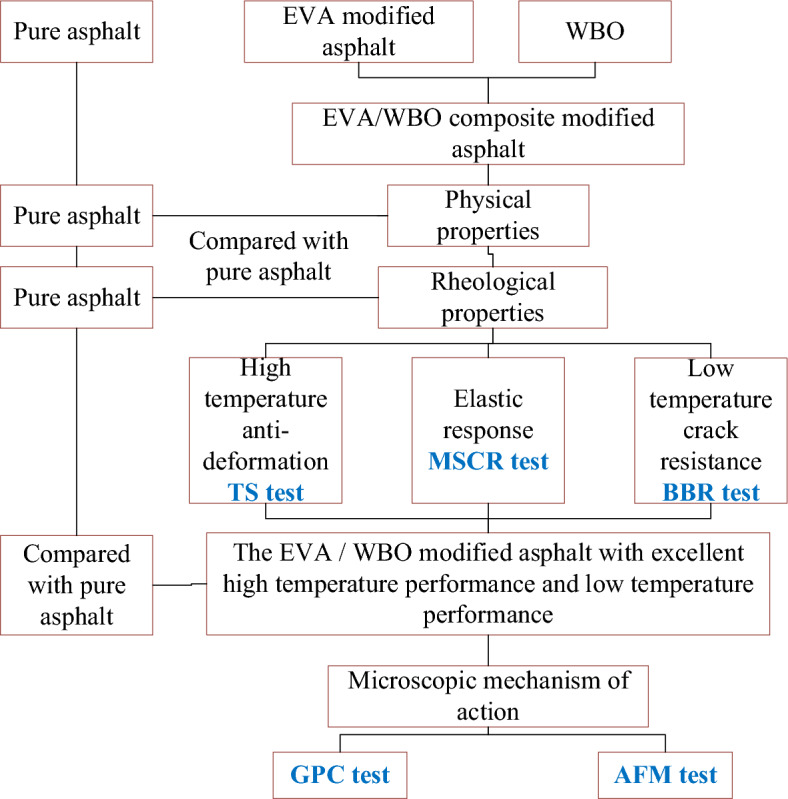
Experimental design.

Figure 1 shows the experimental design of asphalt samples in this study.

## Experiment

### The physical properties

Four basic physical routine tests on the prepared asphalt binder are tested, namely penetration test, softening point test, ductility test (10 °C) and viscosity test at 135 °C. The test procedures refer to ASTM specifications^23–26^.

### Temperature sweep test

One of the tests to evaluate the high-temperature rheological properties of asphalt is the Temperature sweep (TS) test. During the test, periodic shear stress is applied to asphalt samples and stress response of asphalt samples are measured. The parameters obtained in this experiment include G* (dynamic shear modulus), *δ* (phase angle) and G*/*Sinδ* (rutting factor), which could help to understand the elasticity, viscosity characteristics, and rutting resistance of asphalt samples. The parallel plate gap and frequency during the experiment were set to 1 mm and 10 rad/s, respectively. The experimental temperature starts at 46 °C and ends at 76 °C. The experimental steps refer to the specification AASHTO T315^27^.

### Multiple stress creep recovery test

The Multiple stress creep recovery test (MSCR) experiment can assess the viscoelastic response of asphalt samples under intermittent loading mode. This experiment can quantify the elastic and viscous property of asphalt at various stress levels (0.1 kPa and 3.2 kPa). The two important parameters obtained in the experimental report are *J*_*nr*_ (irrecoverable creep compliance) and *R* (elastic recovery rate). The parallel plate specifications and testing temperatures used in the experiment are 25 mm and 64 °C, respectively. The steps of the experiment have been described in detail in the specification AASHTO T350^28^.

### Bending beam rheometer test

In this study, the Bending beam rheological (BBR) test was used to quantify the low temperature crack resistance of asphalt materials. The BBR test is to test the low temperature modulus change of asphalt. The test requires that asphalt to be tested must be poured into size 125 mm × 12.5 mm × 6.25 mm beam. The instrument first applies a preload of 35 mN to the asphalt trabecular beam placed on the support, and then performs a formal loading of 980 mN. The test principle is similar to the loading principle of simple support beam. The displacement–time relationship curve of asphalt trabeculam under the load is collected by the sensor, and the low-temperature stiffness modulus (*S*) and creep rate (*m*) are calculated by the Specification AASHTO T 360^29^.

### Gel permeation chromatography test

The effect of modifiers leads to changes in the macroscopic properties of asphalt, which are generally reflected at its microscopic level. Changes in molecular weight are one of the manifestations. Gel permeation chromatography (GPC) is an advanced technology to detect the molecular weight distribution of materials. It has been used for micro mechanism analysis in the field of asphalt research^30^. The principle of GPC test can be roughly summarized as follows. Compounds with bigger molecular weight are filtered out of the chromatographic column, corresponding to the signal appearing earlier in the signal elution time curve. The compounds with low molecular weight are the opposite. This study analyzed the changes in distribution of molecular weight of asphalts using GPC test. The experiment was conducted at room temperature of 25 °C. Tetrahydrofuran (THF) is used as the dissolution phase.

### Atomic force microscopy test

Atomic force microscopy (AFM) is an advanced method for studying and characterizing the microscopic characteristics of materials^31^. It can generate high-resolution topological images of the sample surface, displaying the morphology and nanoscale structural features of the sample surface. In addition, AFM testing has various advantages, including high-resolution image, non-contact measurement, good environmental adaptability. It has been used to study the surface morphology and analyze roughness of asphalt samples. The AFM test in this study was conducted at room temperature, using a Tap300 probe. Tapping mode is used to minimize the impact on the asphalt sample and obtain a high-resolution 3D image. The raw image data measured by AFM was further processed by nanoscope analysis 3.0 software to obtain surface roughness data.

## Results and discussion

### Analysis of tests of physical property

**Figure 2:**
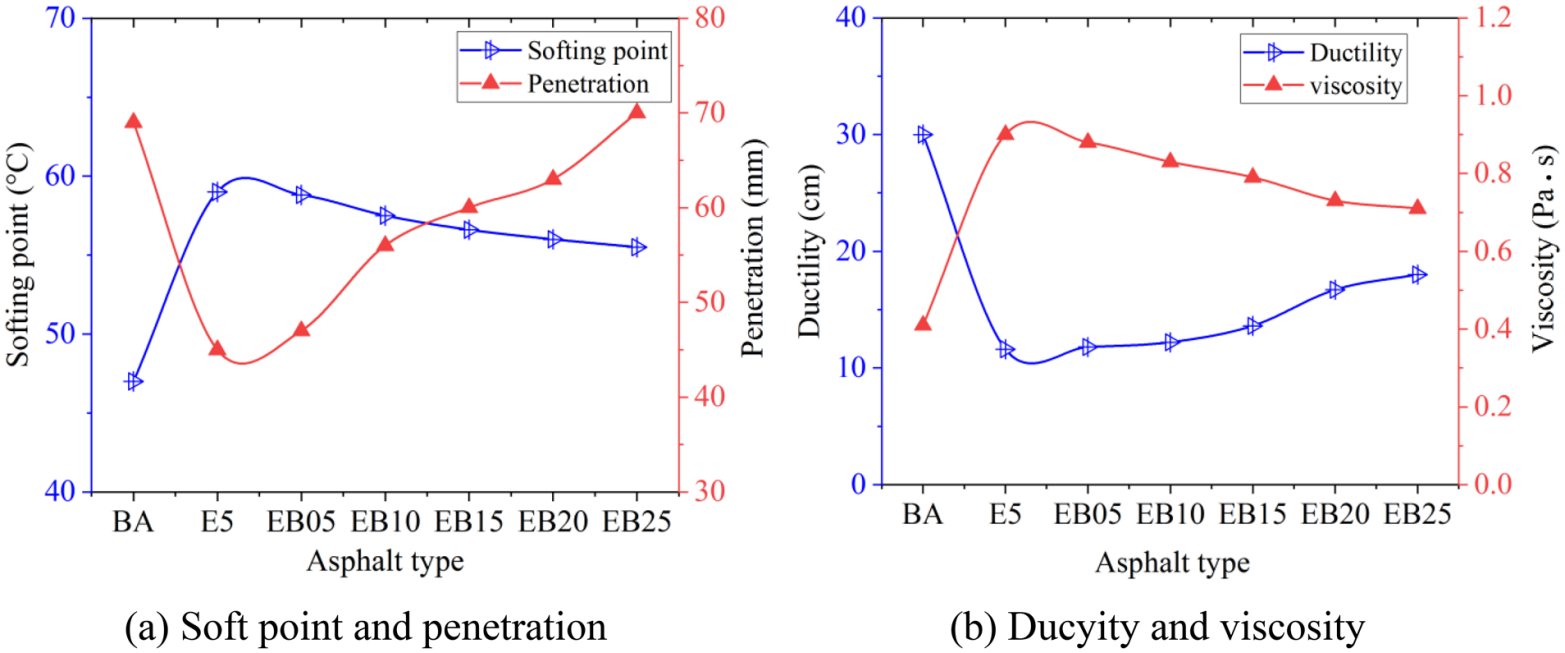
Physical property test results.

The physical property test results of BA, E5 and WEMA samples with various WBO dosages are revealed in Fig. 2. It could be observed that the addition of EVA significantly increases the softening point of the BA, while it significantly reduces its penetration degree. EVA can form a stable network with the pure asphalt, thereby improving its high-temperature performance and stiffness. With the addition of WBO, the softening point of E5 begins to decrease. The softening point of WEMA reaches its minimum value when the WBO content increases to 2.5%. However, it is still significantly greater than the softening point of the BA. As to the penetration, the penetration degree of WEMA increases with the increase of WBO content, indicating that WBO has a softening effect on E5. On the other hand, the ductility of E5 is obviously smaller than that of BA, while the viscosity is significantly bigger than that of BA, and this indicates that EVA has a certain negative impact on ductility at low temperatures and fluidity of BA. After adding WBO to E5, it can be found that WEMA has better ductility and the decreased viscosity, previous research views support this result^32^. This trend develops with the increase of WBO content. This means that WBO has a certain enhancing outcome on the tensile ability of E5 at low temperature, and can also enhance the flowability of E5.

### TS test analysis

**Figure 3:**
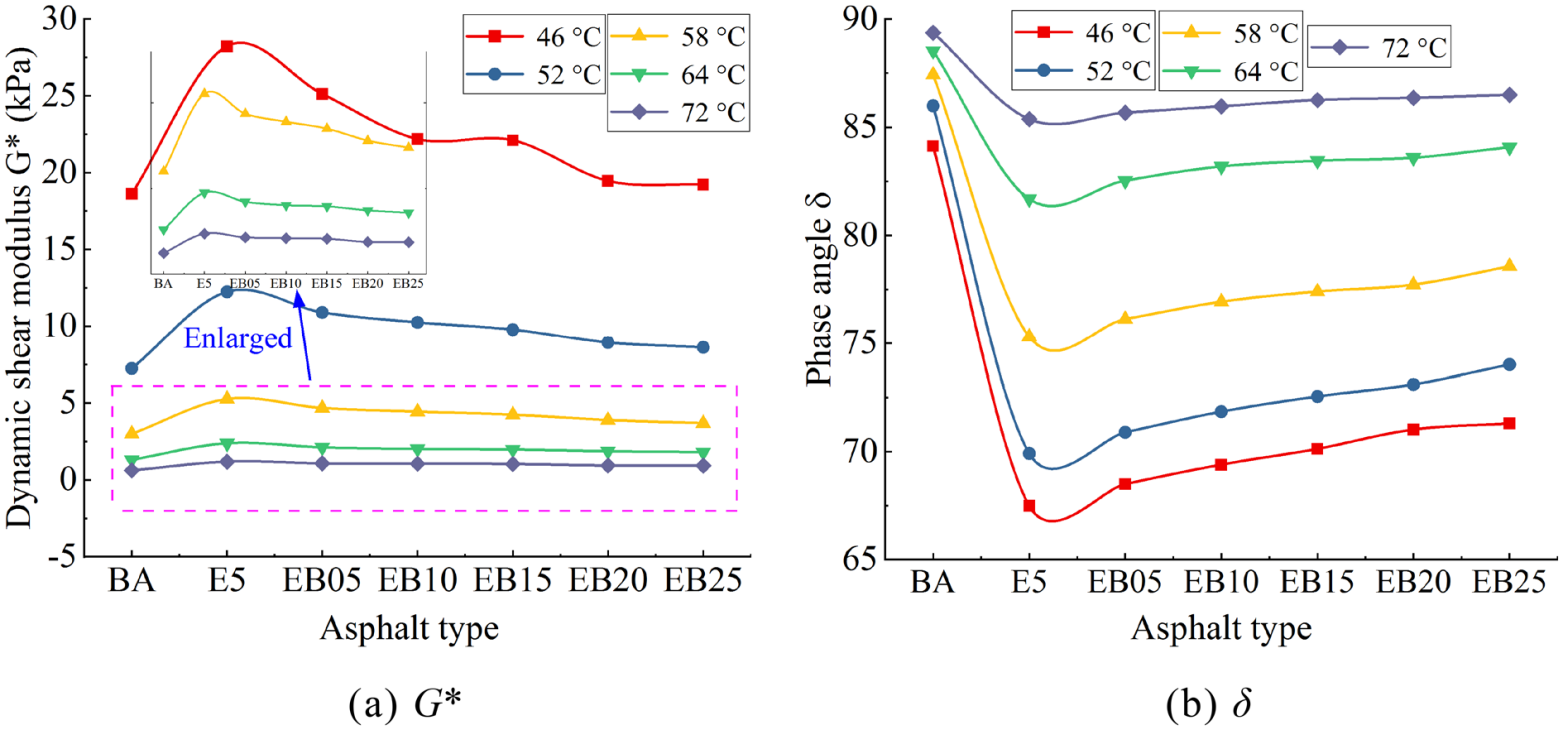
G* and *δ* value.

The G* and *δ* values of all samples are listed in Fig. 3. The G* value of E5 is obviously bigger than the value of BA, and this shows that EVA can enhance the deformation resistance of pure asphalt under high temperature. After adding WBO to E5, the G* value of E5 shows a downward trend when the WBO content increases. Meanwhile, the G* of WEMA reaches its minimum when the WBO content reaches 2.5%. This means that WBO weakens the deformation resistance of E5. On the other hand, *δ* is an indicator for quantifying the viscoelastic response of asphalt^33,34^. When *δ* is smaller, the elastic response of the material is bigger. *δ* value of E5 is significantly smaller than that of the BA, indicating that the elastic response of the pure asphalt under high temperature conditions is enhanced after modification with EVA. The *δ* value of WEMA increases with the increasing content of WBO, which means that WBO has a significant impact on the viscoelastic response of E5. WBO has a negative influence on the E5 sample when it comes to the elastic response, promoting its transition to viscous response, which is consistent with the analysis of WBO’s softening effect on EVA modified asphalt in the penetration results analysis.

**Figure 4:**
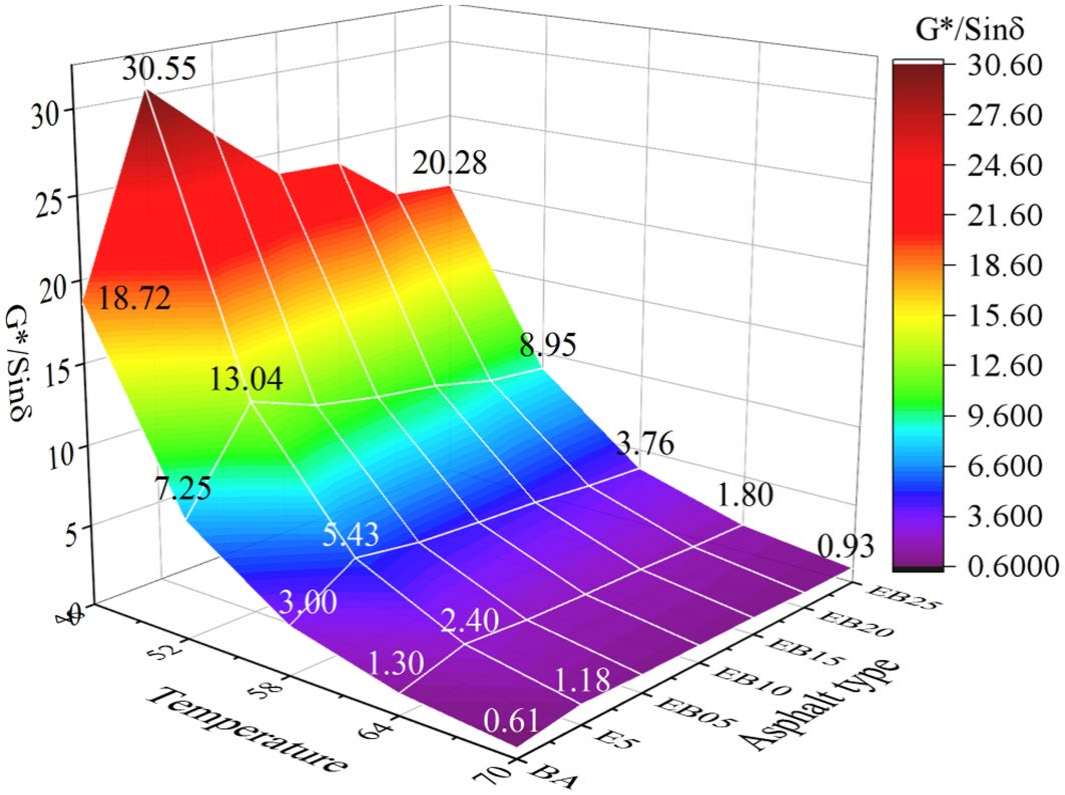
G*/sinδ value.

Figure 4 shows the G*/*Sinδ* values of BA, E5 and WEMA with different WCO dosages. At the same temperature, the trend of changes in G*/*Sinδ* and G* values is similar. The addition of EVA increases G*/*Sinδ* of the pure asphalt. When WBO content increases, its G*/*Sinδ* value shows a downward trend. It could be seen that the G*/*Sinδ* value of WEMA reaches its lowest value at 2.5% WBO content, but it is greater than the test value of the BA under the same conditions. This means that although the addition of WBO weakens the deformation resistance of E5 at high temperature, its high-temperature performance is still significantly better than that of BA. Taking the test values at 64 °C as an example, the G*/*Sinδ* value of EB25 is still 52.46% higher than that of the BA.

### Analysis of MSCR test

**Figure 5:**
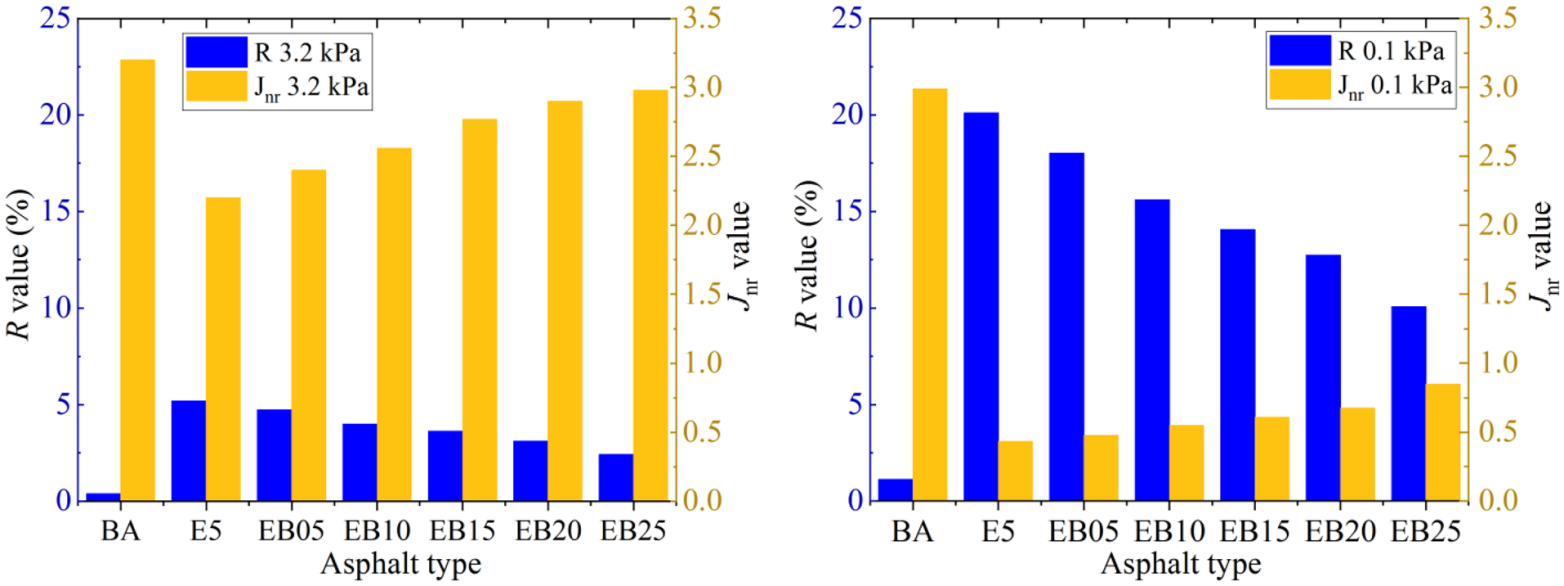
*R* and *J*_*nr*_value.

The test results of *R* and *Jnr* value of all the samples under different stress levels are shown in Fig. 5. The *R* value of E5 is significantly higher than that of BA under different stress loading conditions. This is because the network structure formed by polymer EVA and pure asphalt significantly increases the proportion of elastic response deformation of pure asphalt, and this is consistent with phase angle results in the previous section. In addition, *R* values of E5 under both 0.1 kPa and 3.2 kPa conditions showed a decreasing trend with the addition of WBO. This trend persisted until the WBO content reached 2.5%, indicating that WBO weakened the elastic response of E5 under plastic damage state.

It is worth emphasizing that even when the WBO content is 2.5%, the *R* value of WEMA is still greater than the value of the BA. For the *J*_*nr*_ result, which reflects the irreversible deformation ability, EVA significantly reduces the *J*_*nr*_ value of the pure asphalt at two stress levels. The *J*_*nr*_ value of WEMA increases with the increase of WBO content, and reaches its maximum when the WBO content reaches 2.5%. When *J*_*nr*_ value is bigger, the irreversible deformation caused by the viscosity response is greater, and then the high-temperature deformation resistance is weaker.

### BBR test analysis

**Figure 6:**
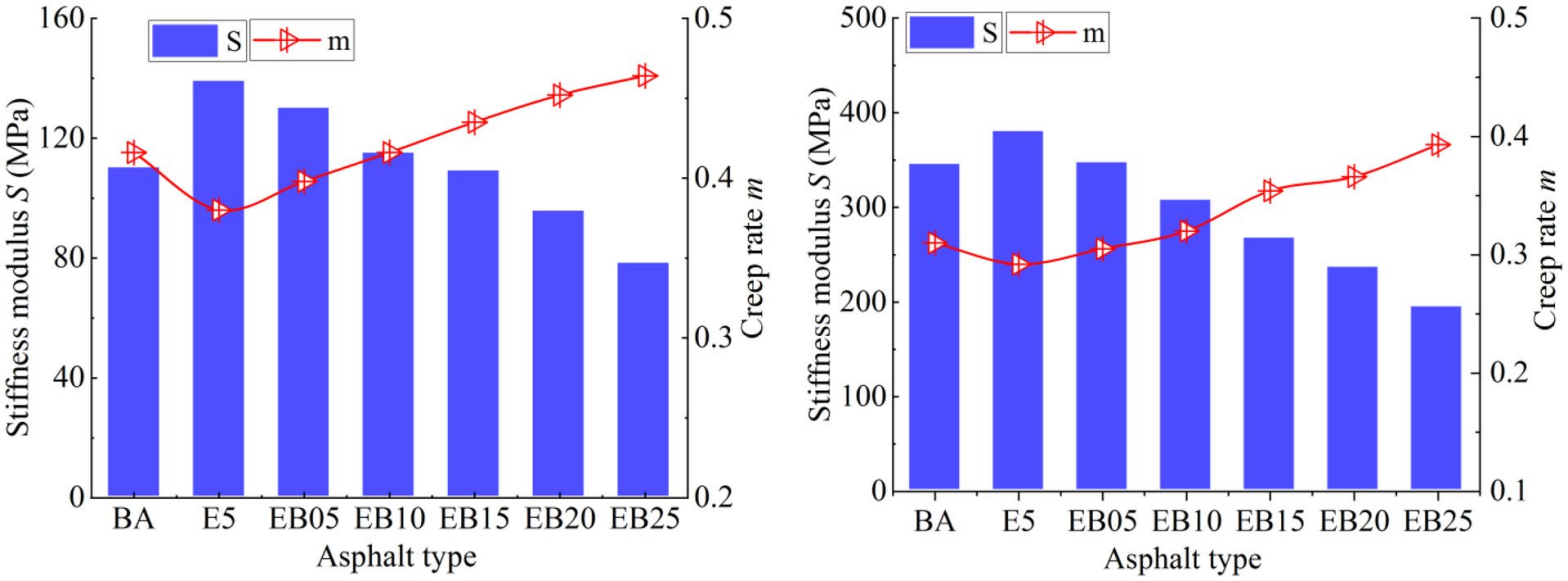
*S* and *m* value.

Value of *S* and *m* test results of all asphalts are shown in Fig. 6. At − 12 °C, the *S* value of E5 asphalt is bigger when compared with the value of BA, and *m* value is lower, indicating that the addition of EVA decreases the property of the pure asphalt when the temperature is low. When WBO is added to E5, the *S* of EVA modified asphalt displays a decreasing trend when content of WBO is from 0 to 2.5%. The change of m value is the opposite.

When WBO content increases, the *m* value gradually increases. It is interesting that when the WBO value is in the range of 1.5–2.5% dosage, its *S* value is already smaller than the value of BA, and its *m* value is higher than that of the BA. A smaller *S* value and a larger *m* value are generally associated with better low-temperature performance. This means that although EVA causes damage to the pure asphalt performance at low temperatures, the modification of WBO compensates for this negative effect and improves the low-temperature property of E5.

At -18° C, influence of WBO on *S* and *m* value of E5 is similar. The increasing of WBO could decrease the *S* value of E5, and *m* increases. When WBO content reaches 1%, the *S* value of E5 is already smaller than that of the BA, and its *m* value is greater than that of the BA; In addition, under the temperature of − 18 °C, 2.5% WBO reduced the *S* value of E5 to 49.2%, which is greater than its reducing extent on the *S* value of E5 under the condition of − 12 °C. This means that WBO has a better and more significant effect on the performance improvement of EVA modified asphalt in lower temperature environments. Other studies have yielded similar views^35^.

### Section summary

Based on the above performance test results, it can be found that EVA significantly enhances the performance of the BA at high temperature, which is in accordance with previous researches. However, the low-temperature performance of E5 decreases. Adding WBO to E5 can weaken its performance at high temperature. Interestingly, the performance of E5 under low temperature is improved after being modified by WBO. It is noteworthy that when the WBO content reaches 1.5%, both the high-temperature and low-temperature performance of EMA are significantly superior to those of pure asphalt. This means that WBO/EVA composite modified asphalt could balance the high and low temperature performance at the same time. To further analyze the mechanism of WBO in EVA modified asphalt, BA, E5, EB05, EB15, and EB25 were selected as samples for studying the microscopic mechanism.

### GPC test analysis

#### Qualitative analysis of chromatograms

**Figure 7:**
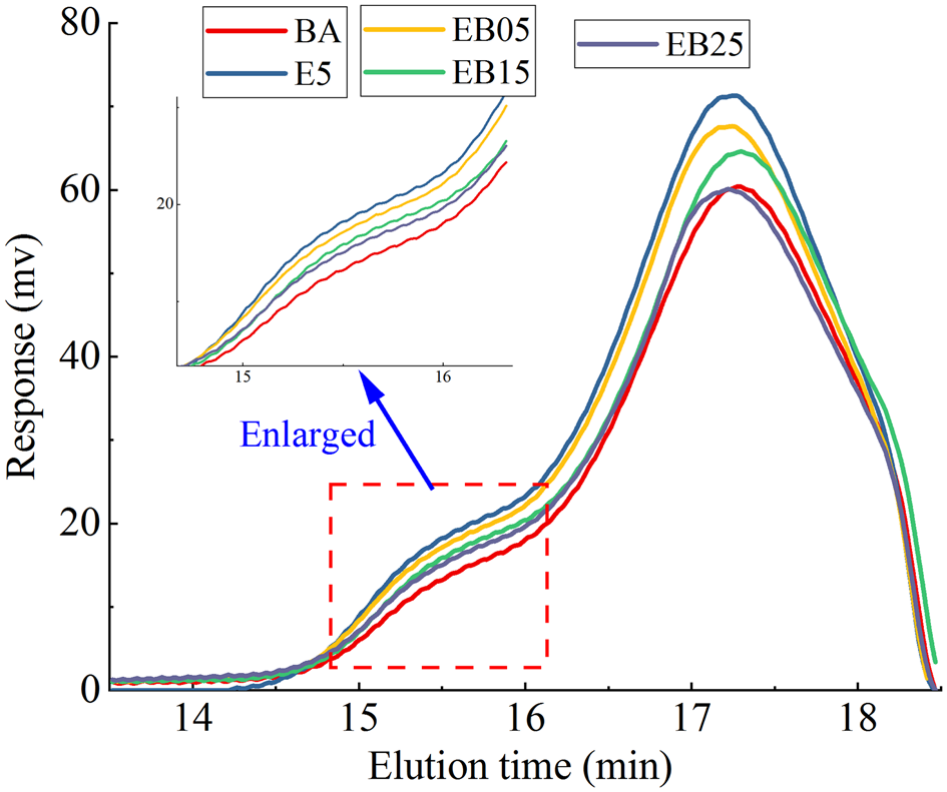
GPC test result.

The GPC test curves of BA, E5 and WEMA are shown in Fig. 7. In GPC testing, substances with high molecular weight are firstly filtered out, which is reflected in the signal peak that appears earlier in the GPC testing curve. The first peak in the asphalt GPC testing curve is generally considered to represent the characteristic peak of large molecular clusters in asphalt^36^. Observing the first peak of BA and E5, it can be seen that the peak of E5 is significantly shifted to the left compared to the pure asphalt. This is due to the modification of EVA, the components in the pure asphalt are cross-linked and swollen with EVA, generating a larger molecular weight macromolecular colloelle material.

After the addition of WBO, the characteristic peak of the macromolecular micelle in E5 shifts to right. The shifting extent of the characteristic peak of the macromolecular micelle in E5 becomes more obvious with bigger WBO content. That is to say that the molecular weight of the macromolecular micelle in WEMA decreases, and this shows that WBO can reduce the molecular weight of the macromolecular micelle of EVA modified asphalt. Then, it could soften the EMA to a certain degree and improve its viscosity deformation response. This result is consistent with the analysis of temperature sweep and MSCR test.

#### Quantitative analysis

The common way for road researchers to process asphalt chromatograms is to divide them into three parts based on the order of elution time, and the order of elution corresponds to the molecular size from large to small. *LMS* (Large molecule size) is considered as an indicator with a outstanding correlation with the property of asphalt, which has been widely recognized in the current researches on asphalt using GPC experiments.

**Figure 8:**
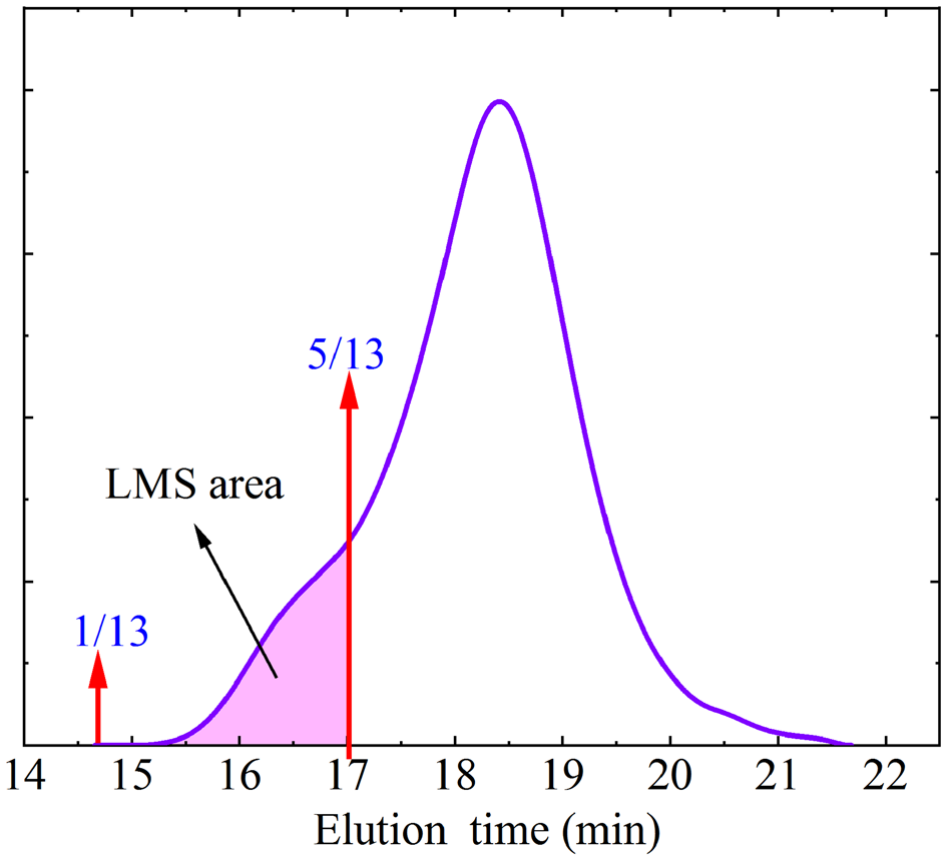
*LMS* diagrammatic.

Referring to the previous analysis method, this section makes the chromatogram be divided into 13 equally spaced intervals, based on the horizontal axis. Intervals of blocks 1–5 are considered as the *LMS* region^37^. Figures 8 shows that the whole area of the chromatogram curve are calculated based on integration. The calculation method of LMS content referring to Eq. (1).

1 $$ LMS(\% ) = \frac{{LMS_{{{\text{Area}}}} }}{A} $$

where *LMS*_Area_ is the *LMS* region area, and *A* is the chromatogram total area.

**Figure 9:**
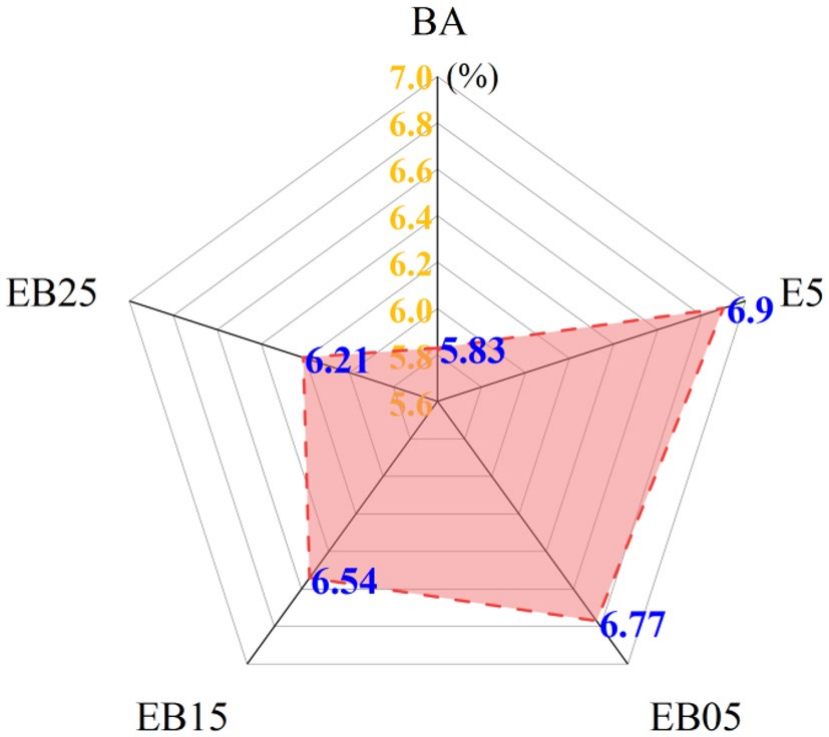
The *LMS* (%) value.

The calculation results of *LMS* value are shown in Fig. 9. Among the five asphalt samples, *LMS* value of E5 is bigger than that of the BA, which shows that under the modification of EVA, the macromolecular micelles increases. Under the theory of asphalt colloid, asphalt is considered to be a colloidal system formed by the dispersion of asphaltene (with relatively high molecular weight) in a medium (with relatively low molecular weight)^38^. The asphaltene is located at the center of the micelle and adsorbs a large number of small molecule light components and soluble media around it. EVA can crosslink with the pure asphalt. The swelling and other reactions generate more stable polymer asphalt macromolecular micelles, so *LMS* can be used to reflect the macromolecular micelles content in the whole modified asphalt. The performance of asphalt at high temperature is often highly correlated with the content of macromolecular micelles in the modified asphalt system. On the other hand, when E5 is treated with WBO, its *LMS* decreases. When 2.5% WBO is added to E5, its *LMS* value decreases by 10%. This means that the content of macromolecular micelles in EMA reduced significantly, and the corresponding macroscopic performance is characterized by the damage to the performance at high temperature, and this is consistent with the analysis of the performance test results in the previous section.

**Figure 10:**
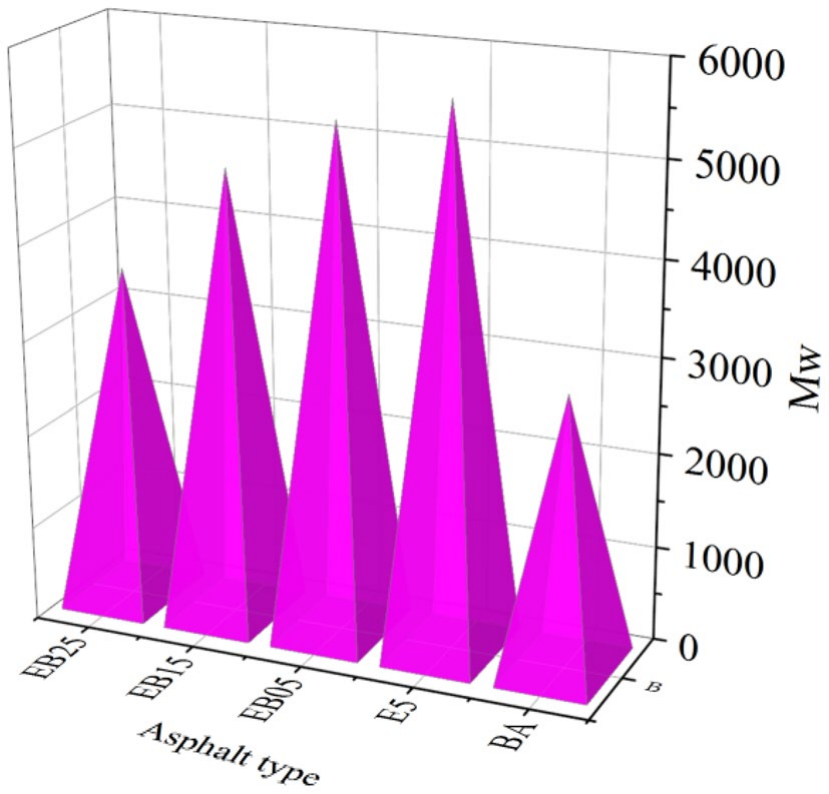
*M*_*w*_ values.

The GPC test also obtained an indicator *M*_*w*_ that reflects the changes in macromolecular micelles. Figure 10 shows that the *M*_*w*_ test results of BA, E5, and WBO/EVA composite modified asphalt. Compared with BA, E5 exhibits a larger *M*_*w*_ value due to the presence of polymers. Moreover, the *M*_*w*_ value of WBO / EVA composite modified asphalt decreased with increasing WBO incorporation, because of the low molecular weight of WBO. When WBO is added to E5, the light components in E5 can be supplemented, thus reducing the molecular weight of macromolecular micelle in of E5 and improving the low temperature crack resistance^39^. Generally speaking, smaller *M*_*w*_ values correspond to weakened performance at high temperature. This is consistent with the trend of the influence of WBO content on the low and high temperature properties of E5 mentioned in the previous section.

### Atomic force microscopy analysis

#### Microscopic morphology analysis

**Figure 11:**
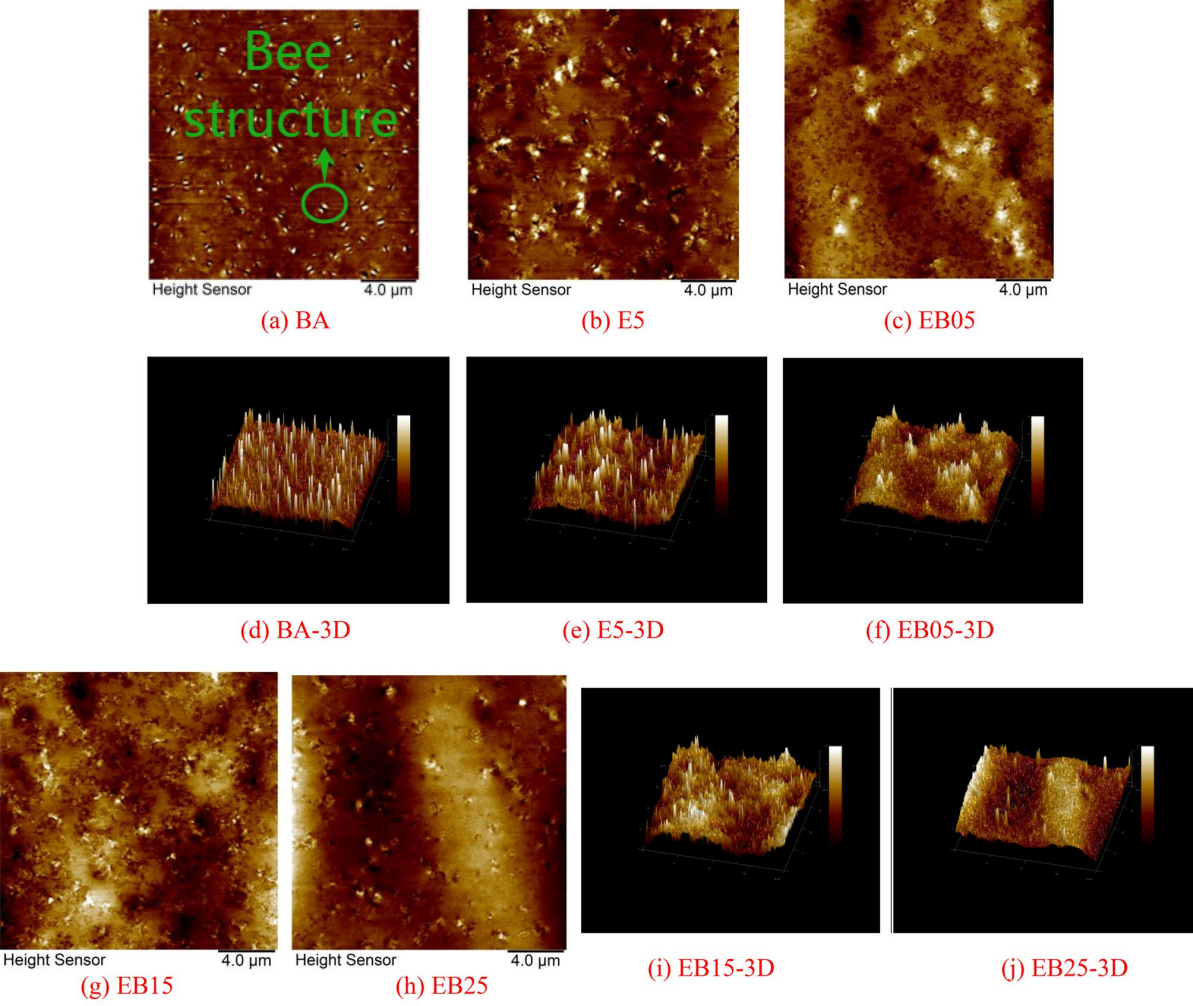
The AFM test images.

The two-dimensional microscopic image of the asphalt sample under AFM test is shown in Fig. 11. The structure composed of the bright and dark areas marked in Fig. 11a is called the “bee structure”. In 3D images, raised peaks correspond to bright areas, and the gaps between adjacent raised peaks correspond to dark areas in 2D images. Currently, most studies believe that asphaltene is the main component of the “bee structure”^40,41^.

The analysis in this section focuses on the differences in the number and morphology of “bee structures” among different testing samples. Comparing E5 with BA, it could be seen that after EVA modification, the “bee structure” in BA changes from a regular strip structure to an irregular shape, and its volume increases. From a quantitative perspective, the number of “bee structures” in E5 is lower than that in BA. This is because the polymer has a large polarity, and EVA polymer can attract each other with the asphaltene components under the action of intermolecular forces, forming a stable cross-linked structure, thus changing the morphology and quantity of “bee structure” in its micromorphology.

Comparing the micro-structure of WBO/EVA composite modified asphalt, it can be found that the “bee structure” of EB05 has evolved into a cluster shape, and the number has significantly decreased. When the content of WBO increases to 1.5% and 2.5%, the cluster like “bee structure” in E5 begins to reduce, and the number of “bee structure” also decreases. Even in EB25, it is difficult to find a more obvious and regular “bee structure”. This can be more intuitively displayed in the 3D microstructure images of E5 and WBO/EVA composite modified asphalt with different dosages. This reveals that WBO can dilute the asphaltene heavy chemical components by supplementing light components, which could induce a reinforcing phenomenon on the property of EVA modified asphalt at low temperature^42,43^.

#### Roughness analysis

**Figure 12:**
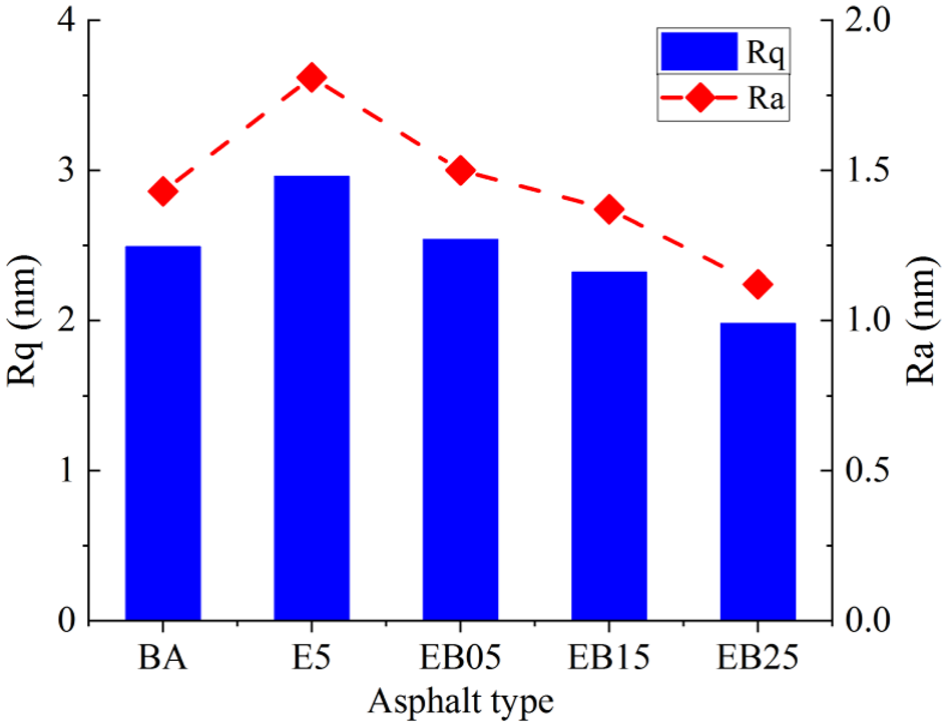
*Ra* and *Rq.*

The AFM of asphalt samples was further analyzed through the Nanoscope Analysis 3.0 software to obtain surface roughness information of different asphalt samples. The roughness parameters include *Rq* (root mean square roughness) and *Ra* (average roughness). The *Rq* and *Ra* values obtained from the AFM tests are shown in Fig. 12. The roughness of asphalt surface at the microscale is related to the physical morphology. When the polymer micelles grow to a certain size, the polymer colloids and asphaltene colloids will come into contact and attract each other^44^. Compared with the pure asphalt, the *Rq* and *Ra* values of the EVA modification are increased, which is obviously caused by the adsorption of EVA polymer to the polar components in the pure asphalt to produce more complex molecular colloids. After adding WBO to E5, the number and volume of asphalt “bee structures” decrease, resulting in a decrease in *Rq* and *Ra* values and a decrease in roughness of EVA modified asphalt. This further reveals the fact that WBO has a softening effect on the EVA modified asphalt .

## Conclusion

To achieve a balance between the low and high temperature performance of EVA modified asphalt, this study prepared WBO/EVA composite modified asphalt (WEMA) using WBO and EVA modified asphalt, and tested its physical properties and rheological properties at low and high temperatures. WEMA having both good low and high temperature performance was obtained, and important modification mechanisms were analyzed through GPC and AFM tests. The conclusions are as follows:

1. The softening point value, penetration and viscosity of EMA decrease when more WBO are used. The ductility of EMA shows the opposite trend, indicating that WBO has a softening effect on EVA modified asphalt material and it weakens its performance at high temperature and improves its tensile performance.
2. Results of TS and MSCR tests indicate that WBO weakens the elastic response and deformation resistance of EVA modified asphalt at high temperature, and it improves the viscosity response of EVA modified asphalt. This result becomes more obvious under bigger WBO content.
3. The BBR results reveals that at − 12 °C and − 18 °C, the effect of WBO can reduce the *S* of EMA and increase *m*. This means that low-temperature rheological properties of EVA modified asphalt are improved by WBO.
4. It is worth noting that even if the maximum content of WBO reaches 2.5%, the performance of WBO/EVA modified materials at high temperature is still more pronounced than that of pure asphalt. When the WBO content reaches 1.5%, the rheological performance of WBO/EVA at low temperature has exceeded that of the pure asphalt. This means that when the WBO content is between 1.5 and 2.5%, the WBO/EVA composite modified asphalt has significantly better low and high temperature rheological properties than the pure asphalt.
5. The analysis of GPC and AFM tests shows that the addition of WBO reduces the content and molecular weight of large molecular groups in EVA modified asphalt, thereby weakening its performance at high temperature. On the contary, WBO dilutes the asphaltene components in EVA modified asphalt by supplementing lightweight components, which plays a positive role for improving the performance of EVA modified asphalt at low temperature.

This study has successfully prepared modified asphalt with excellent high-temperature performance and low-temperature crack resistance using EVA and WBO. This makes it suitable for asphalt road construction in regions with alternating high and low temperatures. For the construction and service of asphalt roads, the anti-aging performance, fatigue performance, and storage stability of EVA/WBO composite modified asphalt need further investigation in future studies.

## Data Availability

The data, models, or code generated or used during the study are proprietary or confidential in nature and can be obtained from the corresponding author upon request.
